# English Review

**Published:** 1891-08

**Authors:** 


### *ENGLISH.*

### The Etiology of Tetanus.

It is now six years since Nicolaier discovered that by inoculating with soil from various sources he could produce typical and fatal tetanus in mice, guinea-pigs, and rodents, and that the seat of inoculation contained in such cases a bacillus with well-defined morphological characters. Subsequent investigation by a large number of observers showed that this same organism is to be found in the pus of the wound in cases of traumatic tetanus of man and the horse, and, although the strict proof that Nicolaier's bacillus is the actual cause of tetanus had not until quite recently been led, there has not for some years past been any doubt on the matter in the minds of most bacteriologists.

If tetanus, quite as much as anthrax or glanders, is a disease caused by the introduction of a micro-organism into the animal body, it is imperatively necessary that the fact should be brought home to human and veterinary practitioners, for an accurate knowledge of the cause of a disease nearly always points to the means of prevention and sometimes also of cure. To veterinary surgeons hitherto the causation of tetanus has generally been regarded as an inscrutable mystery, but, thanks to the researches of bacteriologists, there is no longer anything mysterious about it, as we hope in this article to show.

It has been already indicated that the bacillus discovered by Nicolaier has on many occasions been found cases of natural tetanus; but when it was desired to furnish the experimental evidence necessary to place the etiological *rôle* of the organism beyond any doubt or cavil, considerable difficulty was experienced. To do this it was necessary (1) to cultivate the bacillus in a state of purity outside the animal body, and (2) to re-produce tetanus by inoculating animals with such pure cultures. The difficulty in connection with the first of these steps arose from the fact that the wound which has served as a port of entrance for the tetanus bacillus in cases of traumatic tetanus (natural or experimental) never contains that organism in a state of purity, and, as many of these associated organisms grow in ordinary culture media much more readily than the tetanus organism, the isolation of the latter is not easily effected. But as Kitasato has shown, the tetanus bacillus has two characters by taking advantage of which pure cultures may be obtained from pus containing a variety of other organisms. The first of these is, that it is anærobic, and can therefore be cultivated in media from which oxygen is absolutely excluded. Hence, when a *bouillon* culture inoculated with pus containing a variety of organism besides the tetanus bacillus is incubated in an atmosphere of hydrogen, or *in vacuo*, such organisms as are ærobic do not develop, and a culture is thus obtained containing only the tetanus bacillus and, it may be, one or two other anærobes that were accidentally present along with it in the pus. The second property of which advantage is taken is that of forming spores. The tetanus bacilli after a few days' incubation form spores, which, like the well-known anthrax spores, are vastly more resistant towards heat than the spore-free bacilli, or other organisms in which spore formation does not take place. Accordingly, when a mixed culture obtained by incubation *in vacuo* is heated in a closed vessel for six hours at a temperature of 80° C., the spores of the tetanus bacilli retain their vitality, while all non-sporulating accidental organisms present are killed.

By proceeding in the manner above sketched, Kitasato, Vaillard, Vincent, and others have obtained pure cultures of the tetanus bacillus, and have cultivated them through many successive generations. In such cultures the organism presents itself at first in the form of longer or shorter slender rods, or occasionally as filaments, and in this phase of its development the organ is mobile. At a later stage sporulating bacilli make their appearance; these are slender rods provided at one end with a round refractile spore, which is three or four times as broad as the rod carrying it. A sporulating bacillus thus acquires a striking resemblance to a pin.

We now come to the interesting point of inquiring what effects are produced by inoculation with pure cultures of the tetanus bacillus, and what follows upon that head we condense from a recent article by MM. Vaillard and Vincent,* to whom is due the credit of having cleared up some of the most obscure points in connection with the experimental pathology of tetanus.

The animals found to be most sensible to inoculation with cultures of the tetanus bacillus are white mice, rats, and guinea-pigs; rabbits are also susceptible, but to a less degree, and the dog is very resistant. An extremely small dose of a culture in *bouillon* (1-500 of a cubic centimetre) is sufficient to give typical tetanus to a mouse or a guinea-pig, the symptoms setting in after 12-20 hours, and death resulting in 36-40 hours after inoculation. The rabbit requires considerably larger doses, and the period of incubation is longer and the subsequent course of the disease less rapid. The inoculation succeeds whether the culture be injected under the skin or dura mater, or into the muscles or veins, but infection does not take place by way of the alimentary tract. A guinea-pig can be infected by allowing a few drops of culture to fall on a cutaneous wound on the back, even when that is of small extent and does not intersect more than the superficial layers of the dermis. The symptoms are more or less acute according to the resistance of the animal or the strength of the virus, and actual recovery may take place after 30 days' illness. The symptoms vary also according to the seat of inoculation; the muscles nearest the point at which the culture is introduced first become tetanic, and then the symptoms extend to the adjacent limbs, and finally become generalised.

*Post-mortem* examination of animals killed by inoculation with cultures of the tetanus bacillus reveals only insignificant lesions, such as slight hypæmia or œdema of the subcutaneous tissue, and congestion of the viscera, due to the impeded respiration before death. Moreover, even when death supervenes within 26 hours microscopic examination rarely reveals any bacilli

---

Annales de l'Institut Pasteur, January, 1891.

at the seat of inoculation or elsewhere in the blood or tissues, and this fact is in agreement with what is found to be the case in tetanus occurring naturally (non-experiment) in man and animals.

These facts had been previously ascertained by Kitasato, but it remained for MM. Vaillard and Vincent to point out their correct interpretation. The symptoms which follow inoculation with pure cultures of tetanus bacilli are not due to the growth and multiplication of the bacilli within the body, but solely to a toxic substance or substances contained in the culture at the time of inoculation. The result is thus entirely different from what follows when an animal is infected with a culture of the anthrax bacillus. That this is so is indicated already by the fact that tetanus bacilli are not, or only with difficulty, to be found in the bodies of the animals that have died with the tetanic symptoms, and the complete proof has been furnished by MM. Vaillard and Vincent, who have shown that the same results are obtainable with cultures from which the bacilli have been removed by filtration, and furthermore, that tetanus bacilli or spores when freed from their own toxic products by washing are incapable of producing tetanic symptoms, provided they are inoculated in a state of purity into a healthy tissue.

Last, but not least, MM. Vaillard and Vincent have shown that while the tetanus organism is not pathogenic when inoculated in a state of purity, there is certain circumstances in which it acquires the power to multiply in the animal tissues, and there to generate those toxic substances to the action of which the tetanic symptoms are immediately ascribable. Thus they found that when one inoculates in a guinea-pig ¼-⅓ of a cubic centimetre of a liquid rich in bacilli and spores, but free from toxin (the tetanising substance generated in cultures of the bacillus), the animal does not take tetanus; but if to 1-15 of a cubic centimetre of such a liquid one adds ¼ of a cubic centimetre of a 20 per cent solution of lactic acid, and then injects the whole into the muscles of the thigh, the animal becomes tetanic, the symptoms being exhibited first in the inoculated limb, and then rapidly extending to the other limbs and to the muscles of the neck. The result was the same when the seat of inoculation was injured, not by the injection of lactic acid, but by simple bruising of the part prior to injecting the tetanus germs. Still more interesting from the practical point of view is the discovery that fatal pathogenic properties are conferred upon the tetanus bacilli by inoculating along with them other organisms, or, which comes to the same thing, by depositing the bacilli in an open wound. Into such a wound the everywhere present organisms of suppuration inevitably find their way, and apparently so injure or lower the vitality of the tissues as to enable the tetanus bacilli to multiply, and to generate the fatal toxin.

The whole of these discoveries are in perfect harmony with what was previously known regarding cases of tetanus occurring naturally in men and animals. The tetanus that follows castration, or that which sets in after pricks of the horse's foot, is now easily explained. The tetanus germs, like the common pyogenic organisms, are widely diffused in nature; and the employment of dirty instruments and the neglect of antiseptic precautions in the after treatment of wounds, afford ample opportunity for the occurrence of that mixed infection to which tetanus is due. As is well known, the wound associated with a case of tetanus need not be large, and it is often so small as to escape detection (idiopathic tetanus); but this point also is quite in harmony with the facts brought to light by the researches of Kitasato, Vaillard, Vincent, and others. The previously mentioned toxin is a substance of almost incredible potence. For example, the last two observers obtained cultures so virulent as to be fatal to guinea-pigs in doses not exceeding the 1-1000 of a cubic centimetre, and, of course, the toxin itself formed only a small fraction of this dose.

The moral of these recent discoveries is evident. The practitioner must mentally place tetanus in the list of micro-organismal affections; and when cases of tetanus supervene after castration or other operation, he must not, as many have hitherto done, ascribe them to atmospheric causes. The strict asepsis obtainable in human surgery is difficult—sometimes impossible—to secure in wounds of the lower animals, but the nearest possible approach to it should always be aimed at; and, above all things, care ought to be taken that, through dirty hands or instruments, the operator is not himself the means of infection.—*The Journal of Comparative Pathology and Therapeutics*, June, 1891.

---

### The Discovery of Pleuro-Pneumonia in American Cattle.

Are the lung lesions in contagious pleuro-pneumonia of cattle pathognomonic, or may identical or indistinguishable alterations of lung structure be found in other diseases? This question demands careful consideration, in view of the dispute that has recently arisen between the home authorities and those of the United States regarding the alleged existence of contageous pleuro-pneumonia in United States cattle landed in this country.

At the present time cattle landed in this country from the United States must within ten days be slaughtered at the port of debarkation. The ground for this restriction regarding the importation of United States cattle is the belief that pleuro-pneumonia exists in that country. There are some agriculturists in this country, and, it goes without saying, many more in the United States, who would like to see this restriction removed. The American Government has for some time past been making efforts to stamp out pleuro-pneumonia, and more than twelve months ago it was contended by the Bureau of Animal Industry that these efforts had been successful in eradicating the disease save in a small area around New York City. Notwithstanding that, the British veterinary inspectors continued to find now and again in the lungs of United States cattle landed in this country, lesions which they pronounced to be those of pleuro-pneumonia. These diseased cattle, it was alleged, came from districts which, according to the information possessed by the Bureau of Animal Industry, were free from pleuro-pneumonia.

Two possible explanations of this state of affairs suggested itself: (1) that pleuro-pneumonia might exist in the United States in districts officially regarded as free from the disease, or (2) that the British veterinary inspectors had made a mistake in diagnosis. In order to discover which of these explanations was the correct one, the American Department of Agriculture obtained permission from the British Government to station American veterinary surgeons at the principal seats of slaughter of American cattle in this country, and forthwith three American inspectors took up their residence in London, Liverpool and Glasgow. These officials are charged with the duty of examining the lungs in all cases in which pleuro-pneumonia, or supposed pleuro-pneumonia, is found in American cattle, and by a system of marking and registration they are also supposed to be able to discover the district from which any such diseased animal has been exported.

In the month of April last, some of the animals belonging to a cargo of American cattle landed at Dempford were found after slaughter to present lesions which the British veterinary inspector considered to be those of contageous pleuro-pneumonia. The correctness of this diagnosis was confirmed by the veterinary advisers of the Board of Agriculture in London, but Veterinary Surgeon Wray, the American inspector stationed in London, disputed the accuracy of the diagnosis, and maintained that the lesions in question were not those of pleuro-pneumonia. Portions of diseased lung from one of these animals were submitted to Professors Walley and M'Fadyean, and their opinion was in consonance with that of the home authorities.

In these circumstances, Dr. Wray brought portions of the same lungs to Edinburgh, in order to obtain the opinion of Professor Williams, and, according to information supplied to some of the lay papers, that authority not alone supported the view taken by the American official, but also went so far as to say that the lesions were simply those of broncho-pneumonia, and expressed his "willingness to demonstrate to any body of veterinary practitioners the distinctive features of broncho-pneumonia detected in these American lungs, as distinguished from the characteristic lesions of contagious pleuro."

We understand that portions of these diseased lungs have also been forwarded by the American inspector to the official head of his department in the United States, and some interest will be felt in the attitude taken up in that quarter.

It has, we believe, been generally held in this country that the *post-mortem* diagnosis of contagious pleuro-pneumonia is not a matter of great difficulty to anyone who has had much experience of the disease, that, in fact, an identical pathological picture is never met with any other affection; and it is hardly possible to conceive an explanation of the flagrant difference of opinion that has arisen regarding the present case. If Professor Brown and his assistants at the Board of Agriculture do not know pleuro-pneumonia when they see it, it cannot be from lack of experience; and since the American inspectors were stationed in this country in order to control the diagnosis of the British inspectors, it must be concluded that they were selected from those who had had abundant opportunity to make themselves acquainted with the pathology of pleuro-pneumonia.

It is within the range of possibility that on American soil there exist other bovine diseases having lesions not distinguishable from those of contagious pleuro-pneumonia, and something to this effect was said not long ago by Dr. Salmon, the veterinary adviser to the United States Government. At the Annual Meeting of the United States Veterinary Medical Association, held in Chicago in September last, Dr. Salmon explained the grounds upon which it had been deemed advisable to station American inspectors in this country; and as his statement is in several respects interesting, it may be worth while to quote it here at length. He said, "It seemed very desirable that our Government should have these men there to look after the matter for two reasons:—First, there was a possibility that there had been an error in diagnosis. Those who have had experience with pleuro-pneumonia know how difficult it is to make a diagnosis when you have only one of these animals, and no way of tracing the history of that animal. You may find the condition of the lungs which resembles pleuro-pneumonia, but which also resembles other diseases. We all know that pleuro-pneumonia produces a peculiar effect on the appearance of the affected lungs; but these peculiarities are also found in lungs which have received their irritation from other germs and sometimes by other agents. So that it can hardly be claimed to-day that these symptoms are always those of contagious pleuro-pneumonia. Of course, when you take these symptoms of pleuro-pneumonia and couple them with other characteristics which we generally see, then we begin to have very positive evidence of the diagnosis."*

We need hardly say that we do not quote this passage in order to show what are Dr. Salmon's views regarding the morbid anatomy of pleuro-pneumonia, for if he had any intention of explaining how his subordinates sent over to Great Britain were to distinguish pleuro-pneumonia from other diseases with which British veterinary surgeons had confounded it, his effort was singularly unsuccessful. One thing is made clear, however, viz., that Dr. Salmon considers it difficult to diagnose a case of pleuro-pneumonia, unless the history of the animal is known, and this of course must refer to *post-mortem* diagnosis. We do not share that opinion, and we do not find in the action of Dr. Wray any evidence that he shares it. Be it observed, Dr. Wray did not say with reference to the lungs of the Deptford cattle—"These lesions resemble those of pleuro-pneumonia, but they may be due to something else, and unless you know the history of the animals you have no right to pronounce the disease pleuro-pneumonia;" on the contrary, he appears to have experienced no difficulty in deciding that the lesions were *not* those of pleuro-pneumonia.

---

* Journal of Comparative Medicine and Veterinary Archives, November, 1890, p. 664.

In other words Dr. Wray carries in his mind's eye a picture of what he takes to be a lesion of a pleuro-pneumonic lung, and he did not find it in this case. In the same way, Prof. Brown, Mr. Cope, and the other veterinary officers of the Board of Agriculture also carry with them a mental picture of the appearances of such a lung, and that picture is different from Dr. Wray's. Both cannot be right, and until the American authorities condescend to define those other diseases vaguely alluded to by Dr. Salmon as having lesions analogous to contagious pleuro-pneumonia, they can hardly expect that when British veterinary inspectors in this country encounter in American cattle "the condition of lungs which resembles pleuro-pneumonia, or which also resembles other diseases," they will pass it over, because it may not have been pleuro-pneumonia. In short, if there is a doubt—and probably there was none in this case—home interests must get the best of it.—*The Journal of Comparative Pathology and Therapeutics*. June, 1891.

